# A novel strategy combining Mini-CEX and OSCE to assess standardized training of professional postgraduates in department of prosthodontics

**DOI:** 10.1186/s12909-022-03956-w

**Published:** 2022-12-22

**Authors:** Lin Niu, Yukun Mei, Xiaoqiao Xu, Yi Guo, Zhen Li, Shaojie Dong, Ruirui Liu

**Affiliations:** 1 Key laboratory of Shaanxi Province for Craniofacial Precision Medicine Research, College of Stomatology, Xi’an Jiaotong University, Xi’an, 710004 Shaanxi China; 2 Clinical Research Center of Shaanxi Province for Dental and Maxillofacial Diseases, Xi’an, 710004 Shaanxi China; 3 Department of Prosthodontics, College of Stomatology, Xi’an Jiaotong University, Xi’an, 710004 Shaanxi China

**Keywords:** Mini-CEX, OSCE, Standardization training, Assessment, Professional postgraduates

## Abstract

**Objective:**

Mini clinical evaluation exercise (mini-CEX) and objective structured clinical examination (OSCE) are widely acknowledged as effective measures of resident standardization training (RST) in European and American countries. However, in China primary mini-CEX and OSCE forms are mainly limited in undergraduate clinical examination. Little knowledge is available regarding the validity and right way of mini-CEX /OSCE evaluation system in advanced dental clinical education so far. This study aimed to explore whether combination of mini-CEX and OSCE represents a global-dimension assessment for postgraduate clinical competence in RST.

**Methods:**

Postgraduates who received RST from June 2017 to June 2019 were selected and evaluated by modified mini-CEX/OSCE scales. Each student received evaluations at least twice in the initial and final stages of training (tested every 4 months). A questionnaire was conducted to investigate the satisfaction with the arrangement of RST.

**Results:**

Mini-CEX/OSCE test results indicated that postgraduates have significantly improved their comprehensive competence in RST projects in the department of prosthodontics (*P* < 0.05). Compared to other master of Stomatology students, postgraduates taking up prosthodontics master’s degree have made more progresses through a training period of up to 1 year and four sessions of face-to-face feedback tutoring (*P* < 0.05). Survey results revealed high level of satisfaction on clinical practice evaluation.

**Conclusion:**

Modified mini-CEX/OSCE combined evaluation system is an effective and reliable assessment tool for clinical comprehensive ability in the RST of professional graduates and can fully highlight their respective advantages on the improvement of students’ clinical competency, especially after several rounds of assessments.

**Supplementary Information:**

The online version contains supplementary material available at 10.1186/s12909-022-03956-w.

## Introduction

Resident standardization training is of great significance for medical students to become qualified doctors after graduation. Based on the background of national medical education collaboration, recently the cultivation of professional master’s degree is unified with the standardized training of resident doctors in China, which is called “dual track system”. Postgraduates applying for professional master’s degree are simultaneously required to attend a standardized training program during the postgraduate period. The training of practical skills is dedicated to effectively utilize medical resources and improve the overall quality and level of application-oriented professionals during the three-year limited educational system [1]. However, a series of problems has arose since the implementation of the “dual track system” mode, including internship rotation arrangements, assessment standards, and coordination of clinical and scientific research [2, 3].

Currently, the commonly used clinical skills assessment standards include objective structured clinical examination (OSCE), mini-clinical evaluation exercise (mini-CEX), and direct observation of procedure skills (DOPS). OSCE is a method that can simulate clinical scenes to evaluate the clinical comprehensive ability, understanding and application of theoretical knowledge of students. It is widely used in pre-intern training for specific clinical practice skills of undergraduate students, as well as to motivate students to improve their internship quality. However, the fragmentation of complex clinical cases into brief OSCE stations may result in loss of validity [4]. Mini-CEX is another workplace-based evaluation tool developed by the American College of Internal Medicine to evaluate the clinical ability of residents. It is widely acknowledged for goal-oriented improvement of the comprehensive ability of a resident’s medical skills and humanistic values by timely feedback after test. Furthermore, it can enhance teachers’ sense of teaching responsibility to improve their teaching quality [5]. Since the aforementioned advancement, mini-CEX features a wide range of applications, yet limited information can support its effectiveness in advanced dental education program assessment, especially in the oral prosthodontics, a highly practical subject making the diagnosis and treatment of patients in the department of prosthodontics diverse and complex [6].

Thus, standardizing and strengthening clinical ability training is not only a core requirement but also a challenge in clinical dental education. In view of the characteristics of prosthodontics and the current domestic postgraduate’s training program for professional degree, this study attempted to apply mini-CEX/OSCE scales together to evaluate the clinical training effect on postgraduates by stages in the “dual track system” mode. OSCE assessment was designed to evaluate specific skills in vital tooth preparation for fixed denture using the medical simulator. And the Mini-CEX scale was designed to evaluate the core competencies of postgraduates with a real patient. A questionnaire survey on the satisfaction of standardized training program was also conducted at the end of training, supporting the hypothesis that modified mini-CEX/OSCE combined evaluation system can fully highlight their respective advantages on the improvement of postgraduates’ standardization training and produce “a whole greater than the sum of the parts”.

## Methods

### Study sample

56 postgraduates receiving resident standardization training from July 2017 to June 2019 in the Department of Prosthodontics were selected. All the students had chosen different majors: such as prosthodontics, orthodontics and so on. However, no matter which major they chose, they would be required to participate in standardized training for clinicians, which means that they would participate in clinical training in the department of prosthodontics for at least 4 months. But for postgraduates in prosthodontics, they would spend more time in prosthodontics. They were divided into two groups according to their research direction: prosthodontics group (PG: 27 postgraduates for prosthodontics master’s degree) and non-prosthodontics group (NPG: 29 postgraduates for non-prosthodontics master’s degree). All the subjects only received undergraduate dental clinical practical training before this investigation.

Ethical approval for this study was gained from the Ethical Committee of health science center in our University and written informed consent was obtained from each subjects and patients in the test.

### Assessing process

Based on the domestic and international mini-CEX/OSCE assessment scales and the professional characteristics of prosthodontics, the modified mini-CEX/OSCE feedback scales for dental clinical ability assessment were established by experts in prosthodontic teaching and research section with resident standardized training experience and associated professor title or high position. The assessment criteria and process of the core competence were defined and unified in advance to ensure that the assessment is practical, objective, and fair as much as possible.

Firstly, Mini-CEX scale was designed to evaluate the core competencies of postgraduates with a real patient during training in the department of prosthodontics from eight aspects and develops detailed assessment content for each core competence (Table S1, Supplemental Material). This assessment is a 9-point rating scale organized in three levels: primary level (to be strengthened, 1 ~ 3), medium level (up to standard, 4 ~ 6), and high level (excellent, 7 ~ 9) [5]. Contents of assessment mainly include the common diseases required by the prosthetics syllabus: tooth defect, dentition defect and edentulous; methods of prosthesis involving full crown, inlay, veneer, post-core crown, removable partial denture, and complete denture; basic operation skills of oral restoration including impression taking, tooth preparation, color comparison, recording and transfer of jaw position, denture fitting and bonding.

Secondly, OSCE scale (Table S2, Supplemental Material) was designed to evaluate specific skills in vital tooth preparation for fixed denture using the medical simulator. The assessment contents were evaluated from six aspects including operation processes during tooth preparation. OSCE is a 5-point scale, with a total score of 30 points (total score ≥ 12 is up to standard, ≥18 is good, ≥24 is excellent).

OSCE assessment was designed to evaluate specific skills in vital tooth preparation for fixed denture using the medical simulator. And the Mini-CEX scale was designed to evaluate the core competencies of postgraduates with a real patient. These two evaluation methods were used at the same time to examine the medical ability of postgraduates in medical simulator and actual clinic at the same time.

The clinical ability of postgraduates was evaluated with modified mini-CEX/OSCE scales by stages (tested every 4 months), that is, each postgraduate received at least two evaluations in the initial and final stages of training in the department of prosthodontics (postgraduates in the PG group were required training for 1 year and tested 4 times in total; postgraduates in NPG group were required training for 4 months and tested twice). The entire assessment and evaluation process followed the principle of “separation of teaching and examination.” Three experts were required to attend each assessment and randomly selected cases in the department of prosthodontics according the syllabus. Different experts were required to rate several clinical encounters of a trainee throughout the course rather than a single occasion observed by one individual assessor. After the assessment and evaluation, the assessors would provide face-to-face feedback on the performance of the tested postgraduates and the evaluation results to clinical practice supervisor.

After the completion of the standardized training program, the students will participate in a questionnaire survey (Table S3, Supplemental Material) on the satisfaction of standardization training program in the department of prosthodontics. Please check the supplementary material for more details about the OSCE, the Mini-CEX scale and the questionnaire survey.

### Evaluation of reliability

In order to prove that the two evaluation methods (OSCE&MINI-CEX) used in this experiment have good reliability, we introduce the Cronbach’s alpha as the evaluation standard. The calculation method is as follows, where k is the number of questions in each scale, S_i_^2^ is the variance of the score of each question, and S_x_^2^ is the variance of the total score of the scales.

$$alpha=\left(\frac{k}{k+1}\right)\left(1-\frac{\sum_{i=1}^k{S}_i^2}{S_x^2}\right)$$

All operations were carried out in the same skill operating room with OSCE scale to evaluate specific skills in vital tooth preparation. In the process of OSCE scale, all the treatments were performed in the same dental chair in the prosthodontics department. Therefore, there was no interference due to different locations. In addition, the experts involved in the scoring remain the same, and the scoring criteria are formulated uniformly before the scoring to avoid the bias caused by different raters each time. In the process of using the Mini-CEX scale, the patients were selected from the patients who came to the department of prosthodontics that day, the patients received by each student were random. Although the types of diseases received by each student may be different, they all belong to the scope of diagnosis and treatment of the department of prosthodontics, which will comprehensively examine their ability to deal with different diseases. Before receiving treatment, each patient has been informed that the postgraduates will receive the patient, and all patients have expressed understanding and cooperation. There was no impact caused by the patient’s non-cooperation.

### Statistical analysis

All data were analyzed by SPSS 18.0 statistical software. Repeated measurement analysis was used to test the training effect between the two groups at different stages. The satisfaction degree (percentage) was compared by χ2 test. *P* ≤ 0.05 was considered statistically significant.

## Results

Each assessment in this study was completed within 45 mins. All assessors gave a real-time feedback to the tested postgraduates, and the feedback lasted for an average of 5.6 min. The completion rate of the entire evaluation process is 100%.

The Mini-CEX scale was used 4 times to evaluate the core competencies of postgraduates with a real patient (test 1–4). 8 tests were conducted. OSCE scale was used 4 time to evaluate specific skills in vital tooth preparation for fixed denture using the medical simulator (test5–8). We refer to the non-standardized form to calculate the alpha (Table 1). The Cronbach’s alpha was greater than 0.7 in each test, which confirmed that both the OSCE scale and the Mini-CEX scale used in this experiment had excellent reliability.

**Table 1:** Cronbach’s alpha in each test

Mini-CEX scale		OSCE scale	
Test	Alpha	Test	Alpha
1	0.8298	5	0.7245
2	0.9311	6	0.9051
3	0.9251	7	0.7400
4	0.8260	8	0.8422

**Fig. 1:**
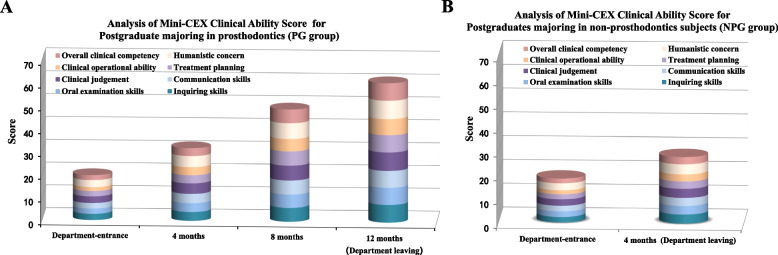
Analysis of Mini-CEX clinical ability score. **A** Stacking histogram of mini-CEX scores in PG group; **B** stacking histogram of mini-CEX scores in NPG group. Note: Although there were no significant differences between PG and NPG groups at the initial stage (*P* > 0.05), statistically significant differences in both single content score and total score of mini-CEX were observed after training at different stages (*P* < 0.05)

The results of the mini-CEX assessment was showed in Fig. 1. The initial scores of entrance examinations were low for both groups. The performance of most postgraduates was “to be strengthened (score ≤ 3)” in most aspects including clinical diagnosis, treatment planning, clinical operation and overall competency. After the training, the competence of postgraduates in two groups has significantly improved compared with the early stage of training in relative projects (*P* < 0.05). Compared with NPG group who were required training for 4 months and tested twice, postgraduates in PG group have made more progresses through a training period of up to 1 year and 4 times of face-to-face feedback tutoring (*P* < 0.05) (Table 2). The percent of up to standard in NPG group was less than 100% after 4 months training, and whereas 100% of those in PG group was approached after 8 months of training. Projects, such as medical reviewing, communication skills, treatment plan and clinical operational ability, have achieved an excellent rate of more than 85% when the training program ended at 12 months; this phase was significantly improved from the previous stages.

**Table 2:** Comparison of the mini-CEX scores in different stages [n(%)]

Assessment items	Percent of up to standard						Percent of excellent					
	Department-entrance		4 months		8 months	12 months	Department-entrance		4 months		8 months	12 months
	PG	NPG	PG	NPG	(PG)	(PG)	PG	NPG	PG	NPG	(PG)	(PG)
**Medical reviewing skills**	**5 (18.52%)**^**A**^	**4 (13.79%)**^**A**^	**20 (74.07%)**^**B**^	**19 (65.52%)**^**B**^	**27 (100%)**^**C**^	**27 (100%)**^**C**^	**0**	**0**	**9 (33.33%)**^**a**^	**8 (27.59%)**^**a**^	**18 (66.67%)**^**b**^	**27 (100%)**^**c**^
**Oral physical examination skills**	**8 (29.63%)**^**A**^	**6 (20.69%)**^**B**^	**21 (77.78%)**^**C**^	**18 (62.07%)**^**D**^	**27 (100%)**^**E**^	**27 (100%)**^**E**^	**0**	**0**	**8 (29.63%)**^**a**^	**8 (27.59%)**^**a**^	**17 (62.96%)**^**b**^	**27 (100%)**^**c**^
**Clinical judgement**	**6 (22.22%)**^**A**^	**5 (17.24%)**^**A**^	**22 (81.48%)**^**B**^	**16 (55.17%)**^**C**^	**27 (100%)**^**D**^	**27 (100%)**^**D**^	**0**	**0**	**9 (33.33%)**^**a**^	**6 (20.69%)**^**b**^	**16 (59.26%)**^**c**^	**23 (85.19%)**^**d**^
**Communication skills**	**8 (29.63%)**^**A**^	**8 (27.59%)**^**A**^	**19 (70.37%)**^**B**^	**18 (62.07%)**^**B**^	**27 (100%)**^**D**^	**27 (100%)**^**D**^	**0**	**0**	**10 (37.04%)**^**a**^	**7 (24.14%)**^**b**^	**20 (74.07%)**^**c**^	**26 (96.30%)**^**d**^
**Treatment plan**	**5 (18.52%)**^**A**^	**4 (13.79%)**^**A**^	**20 (74.07%)**^**B**^	**16 (55.17%)**^**C**^	**27 (100%)**^**D**^	**27 (100%)**^**D**^	**0**	**0**	**10 (37.04%)**^**a**^	**6 (20.69%)**^**b**^	**18 (66.67%)**^**c**^	**23 (85.19%)**^**d**^
**Clinical operating ability**	**5 (18.52%)**^**A**^	**4 (13.79%)**^**A**^	**19 (70.37%)**^**B**^	**15 (51.72%)**^**C**^	**27 (100%)**^**D**^	**27 (100%)**^**D**^	**0**	**0**	**8 (29.63%)**^**a**^	**4 (13.79%)**^**b**^	**16 (59.26%)**^**c**^	**20 (74.07%)**^**d**^
**Humanistic concern**	**7 (25.93%)**^**A**^	**8 (27.59%)**^**A**^	**21 (77.78%)**^**B**^	**18 (62.07%)**^**C**^	**27 (100%)**^**D**^	**27 (100%)**^**D**^	**0**	**0**	**9 (33.33%)**^**a**^	**5 (17.24%)**^**b**^	**19 (70.37%)**^**c**^	**25 (92.59%)**^**d**^
**Overall clinical competency**	**6 (22.22%)**^**A**^	**5 (17.24%)**^**A**^	**19 (70.37%)**^**B**^	**16 (55.17%)**^**C**^	**27 (100%)**^**D**^	**27 (100%)**^**D**^	**0**	**0**	**8 (29.63%)**^**a**^	**5 (17.24%)**^**b**^	**17 (62.96%)**^**c**^	**22 (81.48%)**^**d**^

Groups identified by different upper and lower case letters significantly differ in terms of the percentages of standard and excellent performance in each assessment item, respectively (*Note*: *PG* prosthodontics group, *NPG* non-prosthodontics group.) (*P* < 0.05)

**Fig. 2:**
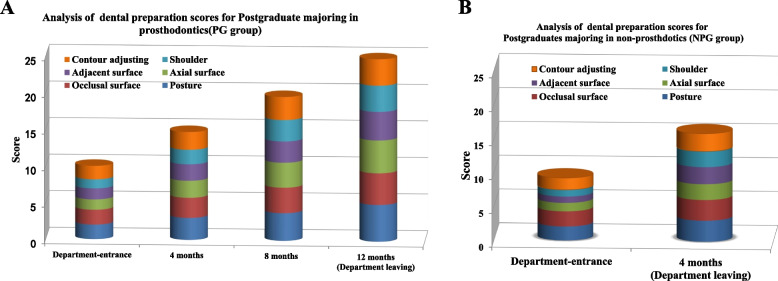
Analysis of dental preparation score (OSCE). **A** Stacking histogram of OSCE score in PG group; **B** stacking histogram of OSCE score in NPG group. Note: Statistically significant differences in both single content score and total score were observed between different stages (*P* ≤ 0.05). However, no significant differences were identified between PG and NPG groups at the same stage (*P* ≥ 0.05)

The OSCE assessment results were shown in Fig. 2. At the beginning of the training, more than half of the students presented an unsatisfactory score in tooth preparations, which indicated the poor ability to control the axial surface degree, preparation amount, and adjustment of the occlusal surface morphology on tooth preparation. After completion of the training, most students on tooth preparation projects were up to standard (Table 3). The postgraduates for prosthodontics master’s degree achieved significantly higher excellent rate of tooth preparation scores after 8 months (*P* < 0.05). The results indicated the tooth preparation project still needs more exercise to be able to make perfect and keep improving. Though the PG and NPG group was not a complete comparison, but it showed that through this training method, no matter whether the postgraduates majoring in prosthodontics or not, they could achieve better results in the clinical training of prosthodontics, and the PG group can achieve better results because of its long participation time.

**Table 3:** Comparison of OSCE evaluation results in different stages [n(%)]

Assessment time	Percent of standard		Percent of good		Percent of excellent	
	PG	NPG	PG	NPG	PG	NPG
**Department entrance**	**10 (37.04%)**^**A,a**^	**10 (34.48%)**^**A,a**^	**0**	**0**	**0**	**0**
**4 months**	**25(92.59%)**^**B,a**^	**23(79.31%)**^**B,b**^	**12 (44.44%)**^**A,c**^	**11 (37.93%)**^**c**^	**3(11.11%)**^**A,d**^	**0**^**d**^
**8 months**	**27(100%)**^**C**^	**–**	**20 (74.07%)**^**B**^	**–**	**12 (44.44%)**^**B**^	**–**
**12 months**	**27(100%)**^**C**^	**–**	**27(100%)**^**C**^	**–**	**21 (77.78%)**^**C**^	**–**

Group identified by different upper and lower case letters are significantly different for columns and rows, respectively (*P* ≤ 0.05)

*Note*: *PG* prosthodontics group, *NPG* non-prosthodontics group

As displayed in Table 4, the survey indicated the satisfaction about training and assessment work. All the students were highly satisfied with the work in the department of prosthodontics and the results of their own training result. They believed that training supervisors had great teaching ability and responsibility, which could give them clear explanation and standardized teaching practice. Most students think that both training work arrangement and mini-CEX/OSCE assessment method is reasonable, comprehensive and easy to perform. The “real-time feedback” allows trainees to recognize and impress themselves with their knowledge gaps.

**Table 4:** Satisfaction survey on the assessment of resident standard training in department of prosthodontics [n (%)]

Investigated items	Very satisfied		Satisfied		Not satisfied		Very dissatisfied	
	PG	NPG	PG	NPG	PG	NPG	PG	NPG
① Whether they are satisfied with the training plan and assessment	**25(92.59%)**	**20 (68.97%)**	**2(7.41%)**	**9(31.03%)**	**0**	**0**	**0**	**0**
② Whether they are satisfied with supervisor’s teaching ability (responsibility, theory, operation, problem, and solving ability)	**26(96.30%)**	**26(89.66%)**	**1(3.70%)**	**3(10.34%)**	**0**	**0**	**0**	**0**
③ Whether the assessors can give a practical, objective, fair assessment and regularly provide face-to-face feedback on performance in each test	**27(100%)**	**28 (96.55%)**	**0**	**1(3.45%)**	**0**	**0**	**0**	**0**
④ Whether supervisors can conduct targeted guidance according to the feedback results of assessment	**27 (100%)**	**27 (93.10%)**	**0**	**2 (6.90%)**	**0**	**0**	**0**	**0**
⑤ Whether the department attaches importance to this training and regularly organizes discussion of medical cases and professional skills learning	**25 (92.59%)**	**27(93.10%)**	**2(7.41%)**	**2 (6.90%)**	**0**	**0**	**0**	**0**
⑥ Whether they are satisfied with the improvement of their practical ability after the end of training	**27(100%)**	**20(68.97%)**	**0**	**9(31.03%)**	**0**	**0**	**0**	**0**

*Note*: *PG* prosthodontics group, *NPG* non-prosthodontics group

Students benefit from improving their weak points of clinical skills under the targeted guidance of a supervisor.

## Discussion

Educational assessment is not only an important reflection of clinical teaching effectiveness, but also a good method to promote the training more efficient. The need for effective assessments has increased and is likely to continue to do so [7]. In the past, the quality assessment of clinical teaching in the department of prosthodontics in our hospital mainly adopted the traditional written test (mainly basic theoretical knowledge) combined with OSCE (mainly assessing basic operational skills). However, OSCE usually uses standardized cases and cannot completely simulate true clinical situations. Thus, OSCE cannot truly and comprehensively reflect the professionalism, flexibility and overall clinical competence of dental residents. With the requirements of professional competence increasingly extending into general practitioners, a more scientific and systematic assessment and evaluation system should be developed to continuously improve the quality of standardized training and students’ comprehensive clinical ability [8].

Mini-CEX is a useful educational instrument for monitoring and fostering the resident’s development with real clinical cases [9]. This assessment presents high application value in dental education by evaluating the clinical core competences of each dental resident in a tabular sub-item. The content of assessment includes not only the residents’ clinical practice skills but also the communication skills between doctors and patients, humanistic care and ability to diagnose and develop appropriate treatment plans. At the same time, timely and effective feedback is obtained, which is important for residents to realize their shortcomings and identify the areas of improvement. Continuously strengthening the advantage of the clinical skills and checking the leakage is suitable for new situations and cultivation of high-quality and comprehensive dental talents. This study modified the traditional examination method by effectively combining OSCE and mini-CEX. The former is responsible for assessing the basic skills in tooth preparation for fixed denture, whereas the latter selects clinical random cases to evaluate the overall clinical competences of trainees during the regular diagnosis and treatment from various aspects. Application of these combined assessments will be conducive to creating a multi-level innovation clinical comprehensive evaluation platform which is practical, objective, and reasonable [10]. The results showed the low qualification rate of the core assessment items in the initial stage of training. For example, the students generally cannot effectively give a complete oral examination which could result in imperfect development of the later treatment plan. In addition, shortage of clinical operating position and insufficient human care were observed. Such result is mainly related to the clinical professional characteristics of prosthodontics. Numerous patients in the department of prosthodontics exhibit complicated conditions in the oral cavity and multidisciplinary treatment is usually required. Therefore, clinicians should not only master solid basic theoretical knowledge and operational skills, but also have the ability to comprehensively analyze and solve complex clinical problems. Moreover, patients in department of prosthodontics present a relatively high proportion of middle-aged and elder, who experience poor vision and hearing and difficulty in communication. Thus, new trainees experience difficulty in effectively and smoothly carrying out the whole medical treatment in the early stage. However, after several months of training, the mini-CEX/OSCE assessment presented the core competences of trainees were significantly improved, consistent with previous research results [11, 12]. The questionnaire survey at the end of training also indicated high satisfaction on the arrangement of resident standardization training including the mini-CEX/OSCE assessment. This result may be related to the following factors: Firstly: mini-CEX/OSCE assessment points are highly targeted and contain the core elements of the diagnosis and treatment abilities of oral clinicians. Thus, the students may easily achieve great progress in the clinical practice [13, 14]. Secondly the assessment teacher conducted “one-on-one” real-time feedback guidance to the trainees. The trainees were more likely to receive detailed guidance of their inadequacies during the diagnosis and treatment process. In addition, “real-time feedback” communication can fully mobilize the enthusiasm of trainees for learning. Moreover, such feedback enables trainees to have a clear target in regular training and effectively improve their clinical weakness. It is the “feedback-improvement-re-feedback-re-improvement” and spiraling process. Thirdly, with the increasing frequency of mini-CEX assessments, a wide range of content and diverse cases can be covered, providing the trainees with the opportunity to follow a number of different special cases and understand the new clinical concepts and technologies, which may stimulate trainees’ interest in oral clinical practice and in training their divergent thinking. It is conducive to comprehensive and objective understanding of the clinical teaching effect of trainees and timely guidance [15]. By combining two different evaluation methods, we examine the postgraduates’ operational skills and actual clinical level, and through the real-time feedback from the experts in the assessment process to make postgraduates know their own advantages and disadvantages, and can timely adjust, check and fill the gaps. This new assessment method will help to improve the level of clinical teaching and improve the operation level and clinical diagnosis and treatment ability of medical students.

In addition, the present study revealed that postgraduates for prosthodontics master’s degree generally received more efficient training than those majoring in other subjects during the same period. The main reason was that the postgraduates majoring in other subjects lacked focus on the clinical training of non-self-majors [16]. Contradictions were also observed between the heavy scientific research tasks and the requirements of clinical standardization training. Thus, the phenomena consisting of emphasis on scientific research and neglect of clinical practice were observed. It suggested that the teaching reform should further improve the postgraduate educational program and enhance the understanding of postgraduate on the regulation policy through active guidance. Moreover, clinical practice is a good opportunity for clinicians to discover medical science problems, so the training of scientific thinking skills in clinical practice must be strengthened in a variety of ways, such as through the new progress reports, reading sessions of literature on clinical cases and so on. The need for improvement on clinical cases discussions and scientific advances is also confirmed during the assessment and questionnaire survey in this study, which set up an interactive feedback between assessor and trainee. Thus, it was a better way to benefit trainees as well as supervisors [17]. Supervisors were always required to improve and perfect the teaching tasks in accordance with timely feedback from the trainees. Even the department will also need adjust the content of clinical internship lectures in time to meet the requirement of trainees on new clinical technologies and promote the continuous improvement of teaching quality. As a result, a win-win improvement of teaching and training, scientific research and clinical ability will be achieved. It is also helpful to cultivate professional talents with high-level medical practical ability, strong humanistic literacy, solid theoretical foundation, strong innovative spirit and lifelong learning ability.

## Conclusion

In view of the characteristics of prosthodontics education, the combination of mini-CEX/OSCE is believed a feasible and effective evaluation tool for prosthodontics clinical education, which can respectively evaluate the operation ability and actual diagnosis and treatment ability of graduate students respectively. Since the unification of professional postgraduate training and standardization of resident doctors is a new breakthrough in the reform of medical education in China, the model of stomatology education needs to be explored continuously, and more effective evidence is needed to support the proposed use or various evaluations, for example, Case Study Teaching Method and DOPS teaching method. This assessment tool allowed for further refinement of educational priorities by highlighting both deficiencies and strengths. Thereafter, it ensured postgraduates attain an acceptable standard to complete the learning of clinical skills and the ability to actually handle patients in the clinic. We hope this tool will help postgraduates have a better understanding of their current strengths and weaknesses. And consequently, reduce adverse event rates during the clinical clerkship. Ultimately, a computerized dental clinical teaching and evaluation system will be suggested. It will greatly reduce the workload of examinations and be environmental protection for paperless. In addition, it will enrich the case data with all kinds of common diseases encountered in clinics in order to promote interdisciplinary integration of stomatology and systematically intensify the development of residents’ comprehensive abilities of clinical analysis and problem solving.

## Supplementary Information

**Additional file 1: Table S1.** Mini-CEX assessment form for department of prosthodontics. **Table S2.** OSCE scale for evaluating specific skills in vital tooth preparation for fixed denture. **Table S3.** Satisfaction survey on the assessment of resident standard training in department of prosthodontics.

## Data Availability

The datasets used and/or analyzed during the current study are available from the corresponding author on reasonable request.
