# Supplementary material for: A novel strategy combining Mini-CEX and OSCE to assess standardized training of professional postgraduates in department of prosthodontics
Source: BMC Med Educ. 2022 Dec 22;22:888. doi: 10.1186/s12909-022-03956-w (PMC9773511; doi:10.1186/s12909-022-03956-w)
Supplement: Supplementary file 1 — Additional file 1: Table S1. Mini-CEX assessment form for department of prosthodontics. Table S2. OSCE scale for evaluating specific skills in vital tooth preparation for fixed denture. Table S3. Satisfaction survey on the assessment of resident standard training in department of prosthodontics. [file 12909_2022_3956_MOESM1_ESM.docx]

**Supplemental Material**

**A Novel Strategy Combining Mini-CEX and OSCE to Assess Standardized Training of Professional Postgraduates in Department of Prosthodontics**

Table S1. Mini-CEX assessment form for department of prosthodontics

| Date: |
| --- |
| Name: Number: Class： |
| Assessor: □Chief Physician □Associate Chief Physician |
| Patient Information: Name: Age:  □Male □Female □First Visit □Subsequent Visit |
| Severity: □Low □Moderate □High |
| Diagnosis: |
| Key point:   □Medical Interviewing Skill □Oral Physical Examination Skills  □Clinical Judgment □Communication Skills  □Professional Attitude □Organizational Effectiveness |
| Assessment Items: |
| Medical Interviewing Skill |
| Score: □1□2□3 □4□5□6 □7□8□9 |
| Oral Physical Examination Skills |
| Score: □1□2□3 □4□5□6 □7□8□9 |
| Clinical Judgment |
| Score: □1□2□3 □4□5□6 □7□8□9 |
| Communication Skills |
| Score: □1□2□3 □4□5□6 □7□8□9 |
| Treatment Plan |
| Score: □1□2□3 □4□5□6 □7□8□9 |
| Clinical Operating Ability |
| Score: □1□2□3 □4□5□6 □7□8□9 |
| Professionalism, Patient Perception |
| Score: □1□2□3 □4□5□6 □7□8□9 |
| Overall Clinical Competence |
| Score: □1□2□3 □4□5□6 □7□8□9 |
| Standard: To be strengthened (1-3) Up to standard (4-6) Excellent (7-9) |
| Time for Test: Time for Feedback: |
| Signature of Trainee: Signature of Assessor: |
| Feedback of Trainee: |
| Comments of Assessor: |

Table S2. OSCE scale for evaluating specific skills in vital tooth preparation for fixed denture

| Name： | | Number: Class： | |
| --- | --- | --- | --- |
| The project appraisal standards | Full Score | Actual Score | Notes |
| Occlusal surface | 5 |  |  |
| Axial surface | 5 |  |  |
| Adjacent surface | 5 |  |  |
| shoulder | 5 |  |  |
| contour adjusting | 5 |  |  |
| Posture | 5 |  |  |
| Time for Test: Signature of Trainee: Signature of Assessor: | | | |

Table S3 Satisfaction survey on the assessment of resident standard training in department of prosthodontics

| Investigated items | Very satisfied | Satisfied | Not satisfied | Very not satisfied |
| --- | --- | --- | --- | --- |
| Whether they are satisfied with the training plan and arrangement |  |  |  |  |
| Whether they are satisfied with supervisor's teaching ability (responsibility, theory, operation, problem solving ability) |  |  |  |  |
| Whether supervisor regularly organize the discussion of medical cases and give a lecture the new scientific progress |  |  |  |  |
| Whether supervisors can conduct targeted guidance according to the feedback results of assessment |  |  |  |  |
| Whether the department attached importance to this training and regularly organizes professional skills learning |  |  |  |  |
| Whether they are satisfied with the improvement of their practical ability after the end of training |  |  |  |  |
